# A *dnaN* Plasmid Shuffle Strain for Rapid *In Vivo* Analysis of Mutant *Escherichia coli* β Clamps Provides Insight Into the Role of Clamp in *umuDC*-Mediated Cold Sensitivity

**DOI:** 10.1371/journal.pone.0098791

**Published:** 2014-06-04

**Authors:** Vignesh M. P. Babu, Mark D. Sutton

**Affiliations:** 1 Department of Biochemistry, School of Medicine and Biomedical Sciences, University at Buffalo, State University of New York, Buffalo, New York, United States of America; 2 Witebsky Center for Microbial Pathogenesis & Immunology, School of Medicine and Biomedical Sciences, University at Buffalo, State University of New York, Buffalo, New York, United States of America; 3 Genetics, Genomics and Bioinformatics Program, School of Medicine and Biomedical Sciences, University at Buffalo, State University of New York, Buffalo, New York, United States of America; University of Massachusetts Medical School, United States of America

## Abstract

The *E. coli umuDC* gene products participate in two temporally distinct roles: UmuD_2_C acts in a DNA damage checkpoint control, while UmuD'_2_C, also known as DNA polymerase V (Pol V), catalyzes replication past DNA lesions via a process termed translesion DNA synthesis. These different roles of the *umuDC* gene products are managed in part by the *dnaN*-encoded β sliding clamp protein. Co-overexpression of the β clamp and Pol V severely blocked *E. coli* growth at 30°C. We previously used a genetic assay that was independent of the ability of β clamp to support *E. coli* viability to isolate 8 mutant clamp proteins (β^Q61K^, β^S107L^, β^D150N^, β^G157S^, β^V170M^, β^E202K^, β^M204K^ and β^P363S^) that failed to block growth at 30°C when co-overexpressed with Pol V. It was unknown whether these mutant clamps were capable of supporting *E. coli* viability and normal *umuDC* functions *in vivo*. The goals of this study were to answer these questions. To this end, we developed a novel *dnaN* plasmid shuffle assay. Using this assay, β^D150N^ and β^P363S^ were unable to support *E. coli* viability. The remaining 6 mutant clamps, each of which supported viability, were indistinguishable from β^+^ with respect to *umuDC* functions *in vivo*. In light of these findings, we analyzed phenotypes of strains overexpressing either β clamp or Pol V alone. The strain overexpressing β^+^, but not those expressing mutant β clamps, displayed slowed growth irrespective of the incubation temperature. Moreover, growth of the Pol V-expressing strain was modestly slowed at 30°, but not 42°C. Taken together, these results suggest the mutant clamps were identified due to their inability to slow growth rather than an inability to interact with Pol V. They further suggest that cold sensitivity is due, at least in part, to the combination of their individual effects on growth at 30°C.

## Introduction

**Figure 1:**
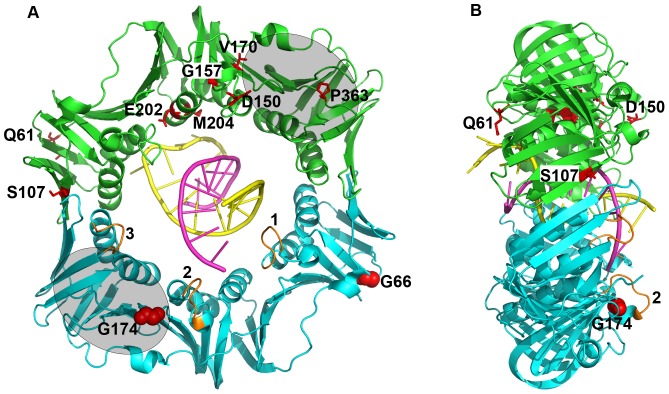
Summary of the positions of β clamp mutations. Shown are (**A**) front and (**B**) side views of the β clamp on DNA (PDB: 3BEP). Amino acid positions bearing substitutions that failed to confer cold sensitive growth when co-overexpressed with Pol V are represented as red sticks in the green clamp protomer. The two residues mutated in the *dnaN159*(Ts) allele (β159; G66→E and G174→A) are indicated as red spacefill in the blue clamp protomer. Loops 1–3 of clamp are higlighted in orange in the blue clamp protomer; loops 1 and 2 contacted DNA in the crystal [5], [6]. The grey ovals represent the approximate location of the hydrophobic cleft present in each clamp protomer that contacts the CBM located in most, if not all clamp partners. This image was generated using PyMOL v1.5.0.2.

The *E. coli dnaN*-encoded β clamp helps to coordinate the actions of several proteins involved in DNA replication, DNA repair and DNA damage tolerance (reviewed in [1]). This essential protein is a head-to-tail homodimer in bacteria (see Fig. 1), the three-dimensional structure and function of which is remarkably well conserved across all domains of life [2]. The β clamp must be loaded onto DNA by a multi-subunit ATPase known as the DnaX clamp loader complex [3], [4]. DnaX opens the clamp at one of the two-dimer interfaces in an ATP-dependent manner [4]. Additionally, loading relies on the ability of both clamp and DnaX to interact in a sequence-independent manner with the DNA template onto which clamp is being loaded [5], [6]. Two of the three loops in the inner core of the clamp, as well as several amino acids lining the inner ring contact DNA during loading ([5], [6]; see Fig. 1). Once loaded onto DNA, several proteins interact with clamp, including all five *E. coli* DNA polymerases (Pols), which rely on clamp for access to the replication fork *in vivo*, as well as processivity (reviewed in [1], [7]). Most, if not all clamp-interacting partners possess a consensus sequence known as the clamp-binding motif (CBM) resembling the QL(^S^/_D_)LF consensus that contacts a hydrophobic cleft located near the C-tail of each clamp protomer ([8]; see Fig. 1). Several important contacts involving surfaces in addition to the CBM-clamp cleft interaction have also been described [6], [9]–[18]. While some of these non-cleft contacts contribute to function of the partner protein when bound to clamp [6], [12], others play critical roles in regulating access of clamp partners to the replication fork [13]–[18].

Following DNA damage, RecA filaments form on single stranded DNA (ssDNA) that accumulates at replication forks due to blocked replication (reviewed in [19]). LexA protein, which acts to repress transcription of >40 unlinked genes, interacts with RecA/ssDNA, resulting in LexA auto-digestion [20], [21]. Auto-digestion serves to inactivate the repressor function of LexA, leading to transcription of the 40+ LexA-regulated genes, also referred to as the SOS regulon [22], [23]. The *polB* (Pol II), *dinB* (Pol IV) and *umuDC* (Pol V) genes are among those regulated by LexA [21]. These Pols possess specialized abilities that enable them to catalyze bypass of DNA lesions that the replicative Pol (Pol III) cannot via a process termed translesion DNA synthesis (TLS; [24]–[27]). Since DNA lesions are often miscoding or noncoding, TLS is often error-prone leading to mutations (reviewed in [1], [19]). The *umuDC*-encoded Pol V is required for most DNA damage-induced mutagenesis in *E. coli* [28], [29]. Following SOS induction, the UmuD and UmuC proteins accumulate, leading to formation of the heterotrimeric UmuD_2_C complex. However, UmuD_2_C lacks detectable Pol activity [30]–[33]. Intact UmuD, together with UmuC, instead acts in a primitive DNA damage checkpoint control [34]. By interacting physically with β clamp, and possibly the α catalytic and ε proofreading subunits of Pol III, UmuD_2_C is suggested to slow Pol III replication in response to SOS induction, thereby allowing additional time for accurate DNA repair functions [35], [36]. TLS requires the UmuD protein to undergo posttranslational modification as part of Pol V licensing [37]–[39]. This process requires RecA/ssDNA, which mediates UmuD auto-digestion (mechanistically similar to LexA auto-digestion), leading to formation of a cleaved form of the *umuD* gene product lacking the N-terminal 24 residues known as UmuD'. Auto-digestion of UmuD to UmuD' serves to release the checkpoint, while simultaneously helping to restart stalled forks by enabling the TLS Pol activity of UmuC [34], [35]. Strains directly expressing UmuC, together with UmuD', were sensitized to killing by ultraviolet light (UV), consistent with the UmuD_2_C checkpoint acting to promote cell survival following SOS induction [34]. Pol II and Pol IV are also suggested to serve checkpoint functions in response to SOS induction by replacing Pol III at the replication fork to slow the rate of replication to permit additional time for accurate DNA repair [17], [40]. Finally, *E. coli* growth was blocked at 30°, but not 42°C, when UmuD_2_C was expressed at ∼6-times the normal SOS-induced level [41]–[43]. In contrast, expression of similar levels of a pre-cleaved form of UmuD (UmuD'), together with UmuC (*i.e*., UmuD'_2_C or Pol V), failed to block growth at 30°C, unless it was overexpressed together with β clamp from a compatible plasmid [44]. Cold sensitivity in both cases was suggested to result from interactions involving β clamp and the different *umuDC* gene products [41].

We previously exploited the cold sensitive growth phenotype conferred by co-overexpression of β clamp and Pol V to identify 8 novel mutant clamps that failed to impede growth at 30°C ([44]; see Fig. 1). Likewise, Beuning and colleagues utilized this same approach to identify 2 mutant UmuD' and 7 mutant UmuC proteins that failed to impede growth at 30°C when co-overexpressed with β clamp [45]. Although the mutant UmuD' and UmuC proteins were not analyzed *in vitro*, the mutant β clamp proteins were [46]. Based on results from *in vitro* solution cross-linking experiments, β^V170M^ and β^P363S^ were weakened for physical interactions with UmuD, while β^G157S^ and β^P363S^ were weakened for interaction with UmuD' [46]. Furthermore, we previously investigated the ability of these mutant clamps, when expressed at physiological levels in a thermolabile *dnaN159*(Ts) strain, to support *E. coli* viability and Pol V mutagenesis. The *dnaN159*(Ts) allele encodes a mutant β clamp protein (β159) bearing G66E and G174A substitutions ([11], [47]; see Fig. 1). All 8 mutant β clamps complemented temperature sensitive growth of the *dnaN159*(Ts) strain, suggesting they retained at least partial ability to support Pol III replication *in vivo* [10], [44]. Moreover, all except β^P363S^ fully complemented the Pol V-dependent mutagenesis defect of the *dnaN159*(Ts) at 42°C, suggesting they were proficient for managing the actions of Pol V [10]. However, despite previous reports suggesting the β159 clamp was inactive at 42°C [48], we recently determined that β159 was able to form functional heterodimers with non-functional clamp proteins *in vivo*, even when strains were grown at 42°C [6], [49]. As a result, it was unknown whether any of the 8 mutant β clamp proteins discussed above were capable of supporting *E. coli* viability and normal *umuDC* functions *in vivo* when expressed as the only clamp protein in the cell.

The goal of this study was to better understand the mechanistic basis of the cold sensitivity conferred by co-overexpression of β clamp and Pol V. We first asked whether *dnaN* mutations identified previously by virtue of their inability to confer cold sensitivity when co-overexpressed with Pol V retained an ability to support *E. coli* viability when expressed as the only clamp in the cell. To this end, we developed a novel *dnaN* plasmid shuffle assay. Using this assay, both β^D150N^ and β^P363S^ were unable to support *E. coli* viability. In contrast, each of the remaining 6 mutant clamps (β^Q61K^, β^S107L^, β^G157S^, β^V170M^, β^E202K^ and β^M204K^) supported viability. We therefore asked whether these mutants supported *umuDC* functions *in vivo*. Based on results of experiments using the plasmid shuffle strains, each of these mutant clamps were indistinguishable from the β^+^ shuffle strain with respect to Pol V-dependent DNA damage tolerance. In light of these findings, which argue that cold sensitivity conferred by co-overexpression of β clamp and Pol V was independent of the ability of clamp to properly manage the actions of the *umuDC* gene products *in vivo*, we analyzed growth of strains that overexpressed either β clamp or Pol V alone. The strain overexpressing wild type β clamp, but not the strains expressing mutant clamps, displayed markedly slowed growth at both 30° and 42°C, while strains overexpressing Pol V (*umuD*'*C*) displayed modestly slowed growth at 30°, but not 42C°. Taken together, these results suggest that cold sensitivity conferred by co-overexpression of β clamp and Pol V results from the combination of their individual effects on growth at 30°C, rather than an ability of these proteins to physically interact in a manner that impedes growth. Our results are discussed in terms of models explaining how overexpression of β clamp and/or Pol V impedes *E. coli* growth.

## Materials and Methods

### Bacteriological techniques

Salient features of the *E. coli* strains and plasmid DNAs used in this study are noted in Table 1. Strains were constructed using P1*vir*-mediated generalized transduction [50], λRed-mediated recombination [51], or CaCl_2_-mediated transformation [52], as detailed in the indicated references. Strains were cultured either in Luria-Bertani (LB: 10 g/l Difco tryptone, 5 g/l Difco yeast extract, 10 g/l NaCl), or in M9 minimal (12.9 g/l Na_2_HPO_4_•7H_2_O, 3 g/l KH_2_PO_4_, 0.5 g/l NaCl, 1 g/l NH_4_Cl) medium supplemented with 0.1 mM CaCl_2_, 2 mM MgCl_2_, 40 µg/ml thiamine, 0.5% glucose or maltose (as indicated), and 0.5% casamino acids or 40 µg/ml each of histidine, arginine, threonine and proline, as indicated. When appropriate, the following antibiotics were used at the indicated concentrations: chloramphenicol (Cam), 20 µg/ml for strains bearing plasmids, and 10 µg/ml for strains bearing the chromosomal *lamB*–His_6_-*dnaN^+^–cat* cassette; tetracycline (Tet), 10 µg/ml for strains bearing plasmids, and 2.5 µg/ml for strains bearing the chromosomal *dnaN–tet–recF* cassette; ampicillin (Amp), 150 µg/ml; kanamycin (Kan), 40 µg/ml; spectinomycin (Sp), 60 µg/ml; and rifampicin (Rif), 50 µg/ml. Oligonucleotides (Sigma or IDT) are described in Table 1.

**Table 1:** *E. coli* strains, plasmid DNAs and oligonucleotides used in this study.

*E. coli* strains		
Strain	Relevant genotype	Source
RW118 a	*lamB^+^ dnaN^+^ lexA^+^ sulA211*	[65]
RW120	RW118: Δ*umuDC595*::*cat*	[65]
MS139	*lamB*::(His_6_ *-dnaN^+^–cam*)	[6]
MS198	RW118: *lamB*::(His_6_ *-dnaN^+^–cam*) *dnaN^+^*	This work
MS199	RW118: *lamB*::(His_6_ *-dnaN^+^–cam*) *dnaN^–1FS^–tet–recF^+^*	This work
MS200	RW118: *lamB*::(His_6_ *-dnaN^+^–cam*) *dnaN^–1FS^–tet–recF^+^* (pAMP*dnaN^+^*)	This work
GS20	*lamB20*::Tn*5*	CGSC b
MS201	RW118: *lamB20*::Tn*5 dnaN^–1FS^–tet–recF^+^* (pAMP*dnaN^+^*)	This work
MS202 c	RW118: *lamB20*::Tn*5 dnaN^–1FS^–tet–recF^+^* (pACM*dnaN^+^*)	This work
MS203 c	RW118: *lamB20*::Tn*5 dnaN^–1FS^–tet–recF^+^* (pACMQ61K)	This work
MS204 c	RW118: *lamB20*::Tn*5 dnaN^–1FS^–tet–recF^+^* (pACMS107L)	This work
MS205 c	RW118: *lamB20*::Tn*5 dnaN^–1FS^–tet–recF^+^* (pACMG157S)	This work
MS206 c	RW118: *lamB20*::Tn*5 dnaN^–1FS^–tet–recF^+^* (pACMV170M)	This work
MS207 c	RW118: *lamB20*::Tn*5 dnaN^–1FS^–tet–recF^+^* (pACME202K)	This work
MS208 c	RW118: *lamB20*::Tn*5 dnaN^–1FS^–tet–recF^+^* (pACMM204K)	This work
AB1157 d	*dnaN^+^ umuD^+^C^+^ lexA^+^*	Lab stock
MG1655 e	*dnaN^+^ umuD^+^C^+^ lexA^+^*	Lab stock

Plasmid DNAs		
Plasmid	Relevant characteristics	Source
pANTF	Kan^R^, Tet^R^; p15A *oriV*; encodes *dnaA^+^–dnaN^+^–tet–recF^+^* cassette	[6]
pANΔXTF	Kan^R^, Tet^R^; p15A *oriV*; encodes *dnaA^+^–dnaN^–1FS^–tet–recF^+^* cassette	This work
pKD46	Amp^R^; pSC101 *oriV* with *repA101*(Ts) mutation; expresses λRed recombinase from the *araBAD* promoter	[51]
pAMP*dnaN^+^*	Amp^R^; p15A *oriV*; expresses physiological levels of β^+^ from the *dnaN^+^* promoter	[10]
pACM	Cam^R^; p15A *oriV*; cloning vector	[10]
pACM*dnaN^+^*	Cam^R^; p15A *oriV*; expresses physiological levels of β^+^ from the *dnaN^+^* promoter	[10]
pACMβ5A	Cam^R^; p15A *oriV*; expresses physiological levels of β^148-152^ from the *dnaN^+^* promoter	[10]
pACMQ61K	Cam^R^; p15A *oriV*; expresses physiological levels of β^Q61K^ from the *dnaN^+^* promoter	This work
pACMS107L	Cam^R^; p15A *oriV*; expresses physiological levels of β^S107L^ from the *dnaN^+^* promoter	This work
pACMD150N	Cam^R^; p15A *oriV*; expresses physiological levels of β^D150N^ from the *dnaN^+^* promoter	[10]
pACMG157S	Cam^R^; p15A *oriV*; expresses physiological levels of β^G157S^ from the *dnaN^+^* promoter	This work
pACMV170M	Cam^R^; p15A *oriV*; expresses physiological levels of β^V170M^ from the *dnaN^+^* promoter	This work
pACME202K	Cam^R^; p15A *oriV*; expresses physiological levels of β^E202K^ from the *dnaN^+^* promoter	This work
pACMM204K	Cam^R^; p15A *oriV*; expresses physiological levels of β^M204K^ from the *dnaN^+^* promoter	This work
pACMP363S	Cam^R^; p15A *oriV*; expresses physiological levels of β^P363S^ from the *dnaN^+^* promoter	This work
pBR322	Amp^R^; ColE1 *oriV*; cloning vector	Lab stock
pJRC210	Amp^R^; pBR322 derivative that expresses β^+^ clamp from the *tac* promoter	Lab stock
pJRCHA-4.1	Amp^R^; pBR322 derivative that expresses β^Q61K^ clamp from the *tac* promoter	[44]
pJRCHA-5.1	Amp^R^; pBR322 derivative that expresses β^S107L^ clamp from the *tac* promoter	[44]
pJRCHA-8.1	Amp^R^; pBR322 derivative that expresses β^D150N^ clamp from the *tac* promoter	[44]
pJRCHA-5G″	Amp^R^; pBR322 derivative that expresses β^G157S^ clamp from the *tac* promoter	[44]
pJRCHA-8I″	Amp^R^; pBR322 derivative that expresses β^V170M^ clamp from the *tac* promoter	[44]
pJRCHA-7.1	Amp^R^; pBR322 derivative that expresses β^E202K^ clamp from the *tac* promoter	[44]
pJRCHA-6F″	Amp^R^; pBR322 derivative that expresses β^M204K^ clamp from the *tac* promoter	[44]
pJRCHA-6.2	Amp^R^; pBR322 derivative that expresses β^P363S^ clamp from the *tac* promoter	[44]
pGB2	Sp^r^; pSC101 *oriV*; cloning vector	[66]
pGY9739	Sp^r^; pGB2 derivative expressing UmuD_2_C from the *umuDC-o* c *_1_* promoter	[57]
pGY9738	Sp^r^; pGB2 derivative expressing UmuD'_2_C (Pol V) from the *umuDC-o* c *_1_* promoter	[57]

Oligonucleotides			
Name	Application	Nucleotide sequence (5′→3′)	Source
DnaNΔXhoI top	Cloning of *dnaN* ^–*1FS*^ allele into pANΔXTF	CCGGTACTGAT*cttag*ATGGAAATGGTGGC f	IDT
DnaNΔXhoI bottom	Cloning of *dnaN* ^–*1FS*^ allele into pANΔXTF	GCCACCATTTCCAT*ctaag*ATCAGTACCGG f	IDT
JK28+2	PCR of *dnaN* ^–*1FS*^ *–tet* from pANΔXTF	CATTGCCAATGCCAACTTTACC	Sigma
RecF back	PCR of *dnaN* ^–*1FS*^ *–tet* from pANΔXTF	CTTCGAATTTTCGTCCGACATGTC	Sigma
DnaA forward	Diagnostic PCR of *dnaN* ^–*1FS*^ allele	CGTAAGATCGAGCAGTTGCGTGAAG	Sigma
R3(R6)	Diagnostic PCR of *dnaN* ^–*1FS*^ allele	TCAGACGCTTCATCGTTGCCTG	Sigma
DnaAP	Diagnostic PCR of *dnaN* ^–*1FS*^ allele	CATGAATGTTTCAGCCTTAGTC	Sigma
RecF bottom	Diagnostic PCR of *dnaN* ^–*1FS*^ allele	CAACGTTTCTCGAGCATTTATACTTGG	Sigma
Beta forward	Sequencing of *dnaN* ^–*1FS*^ *–tet* allele	GACGTTCGCTATTACTTAAATGG	Sigma
Beta reverse	Sequencing of *dnaN* ^–*1FS*^ *–tet* allele	GCCAATCTGTACGCGCAG	Sigma

a The complete annotated genotype for strain RW118 is: *rpsL31 xyl-5 mtl-1 galK2 lacY1 tsx-33 supE44 thi-1 hisG4(Oc) argE3(Oc) araD139 thr-1 Δ(gpt-proA)62 sulA211.*

b CGSC: *E. coli* Genetic Stock Center, Yale University, New Haven, CT 06520, USA.

c These strains were generated by plasmid shuffle; see *Material and Methods* for a detailed description of the *dnaN* plasmid shuffle assay. For strains MS202-MS208, the sequence of each plasmid encoded *dnaN* allele was verified by automated nucleotide sequence analysis, and the –1 frameshift mutation in the *dnaN^–1FS^* allele was confirmed by diagnostic PCR and *Xho*I restriction analysis.

d The complete annotated genotype for strain AB1157 is: *xyl-5 mtl-1 galK2 rpsL31 kdgK51 lacY1 tsx-33 supE44 thi-1 leuB6 hisG4(Oc) mgl-51 argE3(Oc) rfbD1 proA2 ara-14 thr-1 qsr-9 qin-111.*

e The complete annotated genotype for strain MG1655 is: *ilvG rfb-50 rph-1*.

f The sequence corresponding to the *Xho*I restriction endonuclease site (CTCGAG) within the *dnaN* ^–*1FS*^ allele, which contains a C→T substitution and −1 dG frameshift (CTTAG), is shown in lower case italics.

### Construction of the dnaN^–1FS^ strain

**Figure 2:**
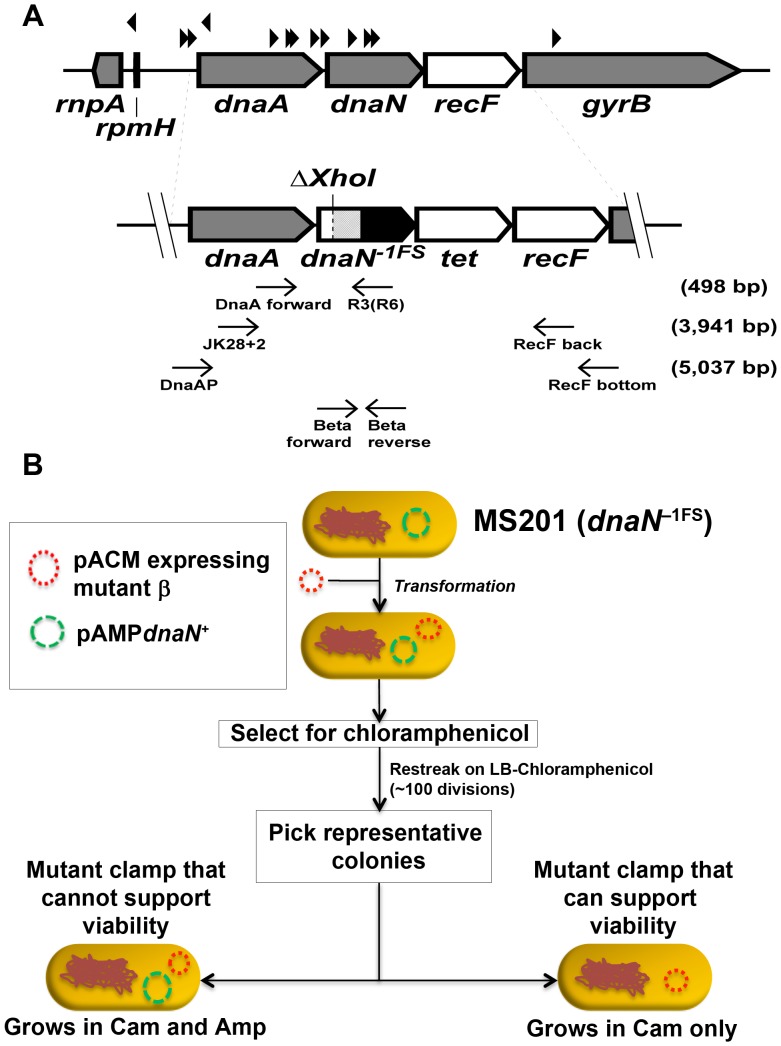
Design of the *dnaN^–1FS^* allele and its use in the plasmid shuffle assay. (**A**) Genomic structure of the *dnaA-dnaN-recF* operon. Genes in grey are essential for cell viability, while those in white are non-essential. Blackened triangles represent approximate positions of confirmed promoters, based on EcoGene 3.0 (http://www.ecogene.org). Gross structure of the *dnaA–dnaN^–1FS^–tet–recF* cassette is depicted below. Δ*Xho*I represents the approximate location of the –1 frameshift mutation present within the *dnaN* ^–*1FS*^ allele. The *dnaN* ^–*1FS*^ allele is predicted to express a protein of 134 residues: the N-terminal 49 residues are identical to the wild-type β clamp protein (white), while the C-terminal 85 residues are distinct and result from the −1 frameshift mutation (light grey). The majority of the *dnaN^–1FS^* allele is not translated (black), due to the premature stop codon at position 135 resulting from the altered reading frame. Relative positions of oligonucleotide primer pairs (see Table 1) used for diagnostic PCR amplification or nucleotide sequence analysis are shown. Expected sizes (in bp) for products of PCR amplified fragments using the noted primer pairs are indicated. (**B**) The MS201 strain contains *dnaN^–1FS^* allele on its chromosome, and bears the Amp^R^ plasmid pAMP*dnaN^+^*, which expresses physiological levels of wild type β clamp that supports viability. After transforming strain MS201 to Cam^R^ with pACM/pACM-derivatives containing the indicated *dnaN* allele, representative pAMP*dnaN^+^* and pACM (or pACM derivative) double transformants are passaged for ∼100 generations before patching onto LB-Amp and LB-Cam plates to score for pAMP*dnaN^+^* retention (*i.e.*, Amp^R^). If the mutant clamp expressed from the pACM plasmid can support viability, pAMP*dnaN^+^* is lost, and cells display an Amp^S^ Cam^R^ phenotype. If the mutant clamp expressed from pACM cannot support viability, the wild type clamp-expressing plasmid pAMP*dnaN^+^* is retained, and cells display an Amp^R^ Cam^R^ phenotype. As controls for strains that readily lost pAMP*dnaN^+^*, we verified the nucleotide sequence of the plasmid encoded *dnaN* allele, as well as the structure of the chromosomal *dnaN^–1FS^* allele (see *Materials and Methods*).

The *dnaN^–1FS^* allele is adjacent to a Tet^R^ cassette (*tet*; positioned between *dnaN* and *recF*; see Fig. 2A) to facilitate selection of recombinants during λRed-mediated recombination, as described previously [6]. The *dnaN^–1FS^* allele expresses a truncated and non-functional clamp protein due to a −1 dG frameshift mutation and a C→T substitution within the *Xho*I restriction site overlapping positions for amino acid residues L49-E50 (Fig. 2A). This allele was introduced into plasmid pANTF using the Quickchange kit (Stratagene) as per the manufacturer's recommendations together with primers DnaNΔXhoI top and DnaNΔXhoI bottom, generating the pANΔXTF plasmid (see Table 1). PCR amplification was for 18 cycles of denaturation (30 sec at 95°C), annealing (30 sec at 55°C), and extension (20 min at 72°C). The sequence of the *dnaA*–*dnaN^–1FS^*–*tet*–*recF* cassette in the pANΔXTF plasmid was verified by automated nucleotide sequence analysis (Roswell Park Biopolymer Facility, Buffalo, NY). The *dnaN^–1FS^*–*tet* cassette was crossed onto the chromosome of strain MS198 using λRed-mediated recombination, as described by Datsenko and Wanner [51]. Briefly, the 3,941 bp *dnaA–dnaN^–1FS^–tet–recF* region was PCR-amplified from pANΔXTF using primers JK28+2 and RecF back (Table 1). The gel-purified fragment was then electroporated into strain MS198 bearing plasmid pKD46, which expresses λRed function under control of the *araBAD* promoter, using a Gene Pulser (2.5 kV, 25 µF, and 200 Ω) equipped with 0.2 cm cuvettes (Bio-Rad). Tet^R^ recombinants resulting from double crossover were selected at 30°C on M9 plates supplemented with 0.5% maltose to allow adequate expression of His_6_-*dnaN^+^* from the *lamB* promoter (*lamB*::(His_6_-*dnaN^+^*–*cam*)), 40 µg/ml of each required amino acid (histidine, arginine, threonine and proline), and 2.5 µg/ml Tet. The correct structure of the recombinant strain was confirmed by diagnostic PCR using primers DnaAP and RecF bottom, and the sequence of *dnaN^–1FS^*–*tet* locus was verified, as described previously ([6]; see Fig. 2A). Plasmid pKD46 is temperature sensitive for replication, due to a *repA101*(Ts) mutation. Consequently, an Amp^s^ isolate (MS199) lacking plasmid pKD46 was obtained by plating at 42°C. Growth of the resulting MS199 strain relied on maltose for expression of the His_6_-*dnaN* allele from the *lamB* promoter. Strain MS201 was constructed by transforming strain MS199 to Amp^R^ with plasmid pAMP*dnaN^+^* (resulting in strain MS200), followed by replacing the *lamB*::(His_6_-*dnaN^+^*–*cam*) allele with *lamB20*::Tn*5* using P1*vir*.

### Plasmid shuffle assay

The plasmid shuffle assay utilizes strain MS201, and is summarized in cartoon form in Fig. 2B. Strain MS201 encodes the *dnaN^–1FS^* allele at the native *dnaN* locus, and expresses physiological levels of β^+^ from the Amp^R^ plasmid pAMP*dnaN^+^*. Strain MS201 was made chemically competent using CaCl_2_ as described previously [52]. The plasmid shuffle was performed by first transforming strain MS201 to Cam^R^ at 37°C with pACM, or pACM plasmids expressing physiological levels of wild type or mutant β clamps. The pACM plasmids and pAMP*dnaN^+^* contain the same p15A origin of replication and are therefore incompatible with each other. Between 2 and 30 randomly selected Cam^R^ transformants were picked and passaged three times on LB-Cam plates (∼100 generations). Between 3 and 5 colony forming units (CFU) from each plate were then tested for the presence of the pAMP*dnaN^+^* plasmid by patching onto LB-Amp and LB-Cam plates. Clones that contained the pACM plasmid expressing the *dnaN^+^* control (pACM*dnaN^+^*), or mutant *dnaN* alleles capable of supporting *E. coli* growth were sensitive to Amp due to the loss of pAMP*dnaN^+^*. For these strains, representative clones were saved after verifying the structure of the chromosomal *dnaN^–1FS^* allele using diagnostic PCR and *Xho*I restriction (see Fig. 2A), as well as the sequence of the plasmid-encoded *dnaN* allele (see strains MS202-MS208 in Table 1). In contrast, the viability of strains bearing *dnaN* alleles unable to support *E. coli* growth (or the pACM control) relied on wild type clamp expressed from pAMP*dnaN^+^*, and thus displayed resistance to both Cam and Amp.

### Susceptibility and mutagenesis assays

Sensitivity to UV was measured by spotting 10 µl of appropriate serial dilutions of overnight cultures onto LB plates. Plates were irradiated with 60 J/m^2^ UV using a germicidal lamp (254 nm; GE Healthcare), then incubated overnight at 37°C prior to imaging. Sensitivity to hydroxyurea (HU; Sigma) was measured by spotting 10 µl of appropriate serial dilutions of overnight cultures onto LB plates containing the indicated concentrations of HU. Plates were imaged after overnight incubation at 37°C.

Ultraviolet light- (UV-) induced mutation frequency was measured using mid-exponential phase cultures resuspended in 0.8% saline to an OD_600_≈1.0. One-ml of each suspension was either exposed or mock-exposed to 50 J/m^2^ UV using a germicidal lamp. One hundred-µl of UV treated or mock treated cells were then inoculated into 5 ml of LB media. After overnight growth at 37°C, 100 µl of each culture was plated onto LB-Rif plates to score for mutants, while appropriate serial dilutions were plated onto LB plates lacking Rif to measure the number of viable cells. UV-induced mutation frequency was defined as the number of Rif^R^ colonies induced by UV minus those observed following mock treatment per 10^6^ viable colonies. The frequency of methyl methanesulfonate- (MMS-; Sigma) induced mutagenesis was determined using mid-exponential phase cultures. Cultures were resuspended in 0.8% saline as described above. One hundred-µl of each normalized sample was added to 5 ml of LB broth containing 1 µl of MMS, and cultures were incubated overnight at 37°C. Mock treated controls were also performed in which MMS was omitted. The following day, appropriate aliquots of each culture were plated onto LB or LB-Rif plates, and MMS-induced mutation frequency was calculated as described above for UV. Standard deviations were calculated using the Student's *t*-test web tool at http://www.physics.csbsju.edu/stats/t-test_bulk_form.html.

### Quantitative western blotting

Overnight cultures of *E. coli* MG1655 bearing either pBR322 or pJRC210 were inoculated into LB-Amp and grown at 37°C with shaking to mid-exponential phase (OD_600_≈0.6). Cells from 1 ml of culture were collected by centrifugation and the cell pellet was resuspended with 40 µl 0.8% saline. Eighty-µl of 4X SDS-PAGE loading buffer (200 mM Tris-HCl (pH 6.8), 8% SDS, 0.1% bromophenyl blue, 40% glycerol, 10% mercaptoethanol) was added, and the mixture was heated to 95°C for 10 min. Ten-µl aliquots of 3 pBR322 control lysates were loaded into the wells of 12% SDS-PAGE gel, as were 10 µl aliquots of 2-fold serial dilutions of the pJRC210 lysate. Proteins were resolved by electrophoresis, then transferred to PVDF membrane (Millipore) using a Trans Blot Turbo semi-dry transfer apparatus (Bio-Rad), and probed overnight at 4°C with anti-β clamp rabbit polyclonal antibodies (1∶50,000) [10]. After washing, goat anti-rabbit secondary antibody (1∶50,000) was applied for 1 hr at room temperature. Immune-reactive material was detected using the Clarity Western ECL Chemiluminescence substrate (Bio-Rad), and was visualized using a ChemiDoc Imager (Bio-Rad). Levels of clamp in each lane were measured using the Image Lab software (Bio-Rad). Values for the pJRC210 samples were plotted versus their dilution factor to verify signals were within the linear range of detection (R^2^ = 0.97). A 9.2 (±0.9)-fold increase in clamp levels for the pJRC210 strain relative to the pBR322 control was calculated by comparing the intensity of chromosomally-expressed clamp in each pBR322 control lysate to the dose curve generated using the serially diluted pJRC210 lysate.

### Measurements of transformation efficiency and colony size

Transformation efficiencies at 30° and 42°C represent averages at least 3 independent determinations for each strain. Colony diameter as a function of temperature and incubation time was measured (in mm) for a minimum of 20 randomly selected representative colonies using Adobe Photoshop CS4 from untouched original images. Standard deviations were calculated using the Student's *t*-test web tool at http://www.physics.csbsju.edu/stats/t-test_bulk_form.html.

## Results

### Residues D150 and P363 of the β sliding clamp contribute to one or more functions required for *E. coli* viability

We previously exploited the cold sensitive growth phenotype conferred by co-overexpression of β clamp and Pol V to identify 8 novel mutant clamp proteins that failed to block growth at 30°C ([44]; see Fig. 1). It was unknown whether these mutant clamps were capable of supporting *E*. *coli* viability and normal *umuDC* functions *in vivo* when expressed as the only clamp protein in the cell. To answer these questions, we developed a novel *dnaN* plasmid shuffle assay (Fig. 2B). Briefly, the *dnaN* gene on the chromosome of strain MS201 (Table 1) was disrupted by a −1 frameshift mutation targeting residue Glu-50 (*dnaN^–1FS^*; see Fig. 2A). As a result, viability of MS201 was dependent on physiological levels of β clamp expressed from the Amp^R^ plasmid pAMP*dnaN^+^* (Table 1). We chose to construct the *dnaN^–1FS^* allele rather than delete the *dnaN* gene because of the large number of *recF* promoters located within *dnaN* ([53], [54]; see Fig. 2A). As supported by results discussed below, as well as those published previously [6], neither the *dnaN^–1FS^* mutation nor the *tet* cassette inserted between *dnaN^–1FS^* and *recF^+^* confer polar effects. Cam^R^ plasmids expressing mutant clamps (pACM and its derivatives) were introduced into strain MS201 by transformation. Since these Cam^R^ plasmids belong to the same incompatibility group as pAMP*dnaN^+^*, the respective ability of each mutant clamp to support viability of *E. coli* was measured by scoring for loss of Amp^R^ conferred by pAMP*dnaN^+^*. If a mutant clamp supported viability of *E. coli*, the plasmid expressing the wild type clamp was lost, resulting in a Cam^R^ strain expressing only the mutant clamp of interest (see Fig. 2B). As controls for this experiment, we used (1) plasmid pACM, which lacks a copy of *dnaN*, (2) plasmid pACM*dnaN^+^*, which expresses wild type clamp, and (3) plasmid pACMβ5A, which expresses a mutant clamp bearing alanines in place of residues H148-R152 ([10]; β^148–152^); importantly, β^148–152^ failed to support *E. coli* viability when crossed onto the chromosome at the *dnaN* locus [6]. As summarized in Table 2, strains bearing either pACM (control) or pACMβ5A (β^148–152^) retained pAMP*dnaN^+^*. These results verify both the essential nature of β clamp, as well as the inability of β^148–152^ to substitute for wild type clamp. In contrast, the strain transformed with pACM*dnaN^+^* readily lost pAMP*dnaN^+^*, as expected since both plasmids express physiological levels of the wild type clamp protein. The strains expressing β^D150N^ or β^P363S^ retained plasmid pACYC*dnaN^+^* (Table 2), indicating these mutant clamps were unable to support growth of *E. coli* when expressed at physiological levels as the sole clamp protein. In contrast, strains expressing β^Q61K^, β^S107L^, β^G157S^, β^V170M^, β^E202K^ or β^M204K^ each readily lost plasmid pAMP*dnaN^+^* (Table 2), demonstrating the ability of these mutants to support viability when expressed as the only clamp protein in the cell. In support of this conclusion, the sequence of each plasmid-encoded mutant *dnaN* allele, as well as the correct structure of the chromosomal *dnaN^–1FS^* locus was verified in representative clones (Table 1; see strains MS202-MS208).

**Table 2:** Ability of mutant β clamp proteins to support viability of *E. coli*.

Transforming plasmid a	β clamp protein being assayed b	Amp^R^ CFU/Cam^R^ CFU after ∼100 generations (frequency of pAMP*dnaN^+^* retention) c	Ability of mutant *dnaN* allele to support *E. coli* viability d
pACM*dnaN^+^*	β^+^ (positive control)	0/116 (<0.86%)	+
pACM	None (negative control)	110/110 (100%)	–
pACMβ5A e	β^148–152^ (negative control)	113/113 (100%)	–
pACMQ61K	β^Q61K^	0/15 (<6.67%)	+
pACMS107L	β^S107L^	0/14 (<7.14%)	+
pACMD150N	β^D150N^	112/112 (100%)	–
pACMG157S	β^G157S^	1/19 (5.26%)	+
pACMV170M	β^V170M^	0/6 (<16.7%)	+
pACME202K	β^E202K^	0/10 (<10.0%)	+
pACMM204K	β^M204K^	1/14 (7.14%)	+
pACMP363S	β^P363S^	110/110 (100%)	–

a See Table 1 for a description of the plasmids.

b Amino acid substitutions are indicated in superscript (*e.g*., Q61K represents a lysine substitution of residue Q61).

c Amp^R^ CFU/Cam^R^ CFU is a direct measure of the fraction of Cam^R^ pACM clones bearing the Amp^R^ pAMP*dnaN^+^* plasmid. It was determined by selecting at random colonies that had been passaged for ∼100 generations on LB-Cam plates and patching them onto LB-Amp and LB-Cam plates. Ratios (Amp^R^ CFU/Cam^R^ CFU) observed for each plasmid are shown, while the % frequency is shown in parentheses. At least 1 representative clone for each Cam^R^ and Amp^S^ strain identified was further characterized to verify the presence of the chromosomal *dnaN^–1FS^* allele using diagnostic PCR and *Xho*I restriction, as well as nucleotide sequence of the plasmid-encoded *dnaN* allele.

d Viability refers to the ability of the Cam^R^ transforming plasmid to support growth of *E. coli* in the absence of pAMP*dnaN^+^*. Symbols are as follows: –, plasmid is unable to support viability of *E. coli*, meaning 100% of the CFUs are resistant to both Amp and Cam after ∼100 generations of growth under selection for Cam^R^; +, plasmid is able to support viability of *E. coli*.

e Plasmid pACMβ5A expresses the β^148–152^ mutant, which contains alanines in place of residues H148-R152 [10]. This mutation failed to support *E. coli* viability when crossed onto the bacterial chromosome [6], and serves as an additional negative control for the plasmid shuffle assay.

### Mutant β clamp proteins support normal *umuDC* functions *in vivo*

**Figure 3:**
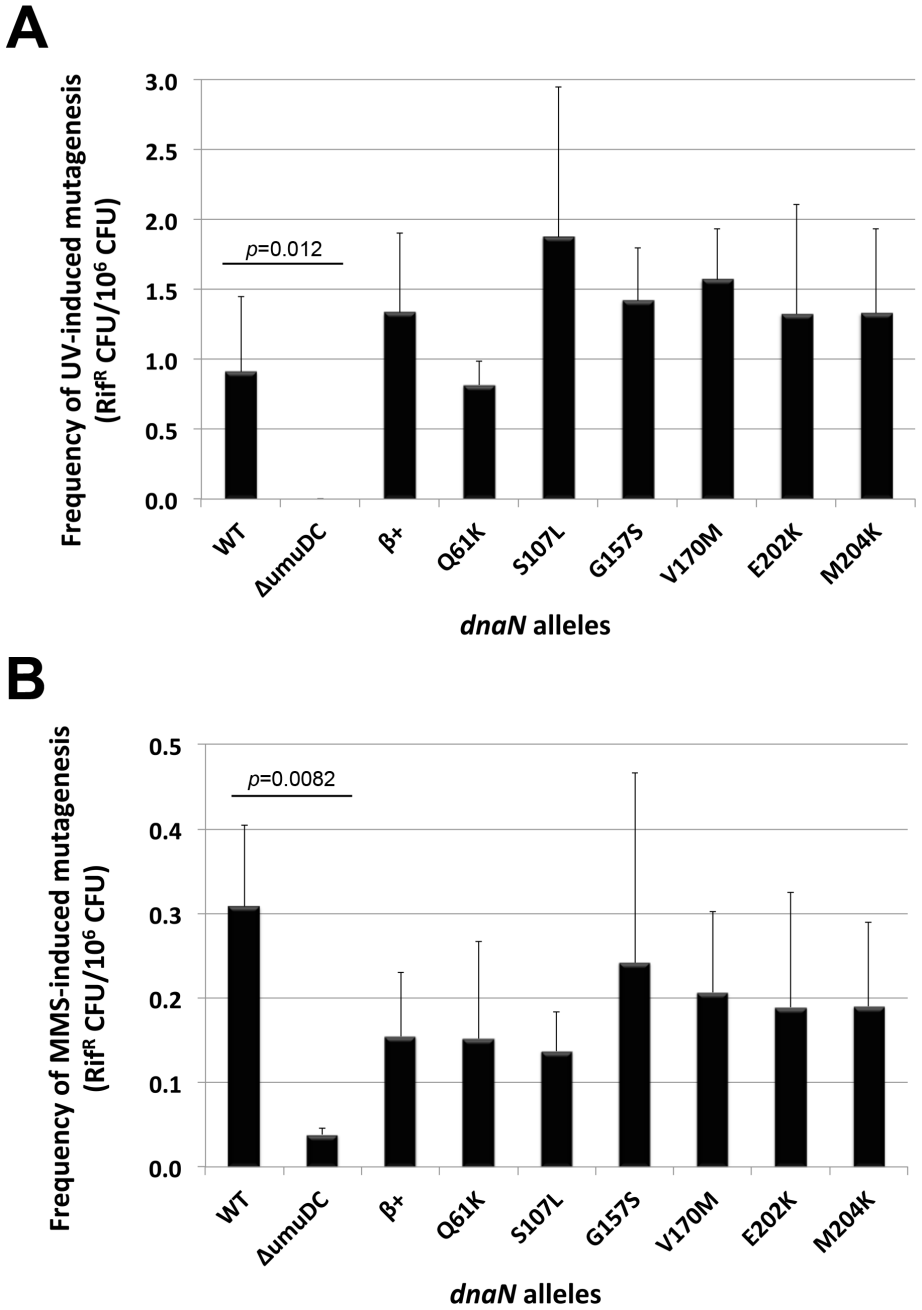
Respective abilities of mutant β clamp proteins to support DNA damage-induced mutagenesis. Frequencies of (**A**) UV- or (**B**) MMS-induced mutagenesis were measured as described in *Material and Methods* using strains RW118 (WT; *dnaN^+^ umuD^+^C^+^*), RW120 (ΔumuD; *dnaN^+^* Δ*umuDC595*::*cat*), or the *umuD^+^C^+^* plasmid shuffle strains MS202 (β^+^), MS203 (β^Q61K^), MS204 (β^S107L^), MS205 (β^G157S^), MS206 (β^V170M^), MS207 (β^E202K^) and MS208 (β^M204K^), as indicated. Results represent the average of 5 independent determinations. Error bars represent one standard deviation. *P*-values ≤0.05 are indicated, and were calculated using the Student's *t*-test.

We took advantage of the strains we made using the plasmid shuffle assay to measure the ability of the mutant clamp proteins to support Pol V function *in vivo*; β^D150N^ and β^P363S^ were not included in this analysis since they failed to support *E. coli* viability (Table 2). Pol V is required for most mutations induced by UV irradiation ([28], [29]; Fig. 3A). Thus, if one or more of the mutant β clamp proteins were impaired for interaction with Pol V, the strain expressing this clamp would display a reduced frequency of UV-induced mutagenesis compared to the β^+^ shuffle strain control (MS202). As summarized in Fig. 3A, frequencies of UV-induced mutagenesis for strains expressing each of the different mutant clamps were indistinguishable from that measured for the β^+^ control. Pol V is also required for most mutations induced by MMS [55]. As summarized in Fig. 3B, each of the mutant clamps was comparable to the β^+^ control with respect to MMS-induced mutagenesis. Taken together, these results indicate that β^Q61K^, β^S107L^, β^G157S^, β^V170M^, β^E202K^ and β^M204K^ are indistinguishable from β^+^ in terms of their respective abilities to manage the actions of Pol V in TLS *in vivo*.

**Figure 4:**
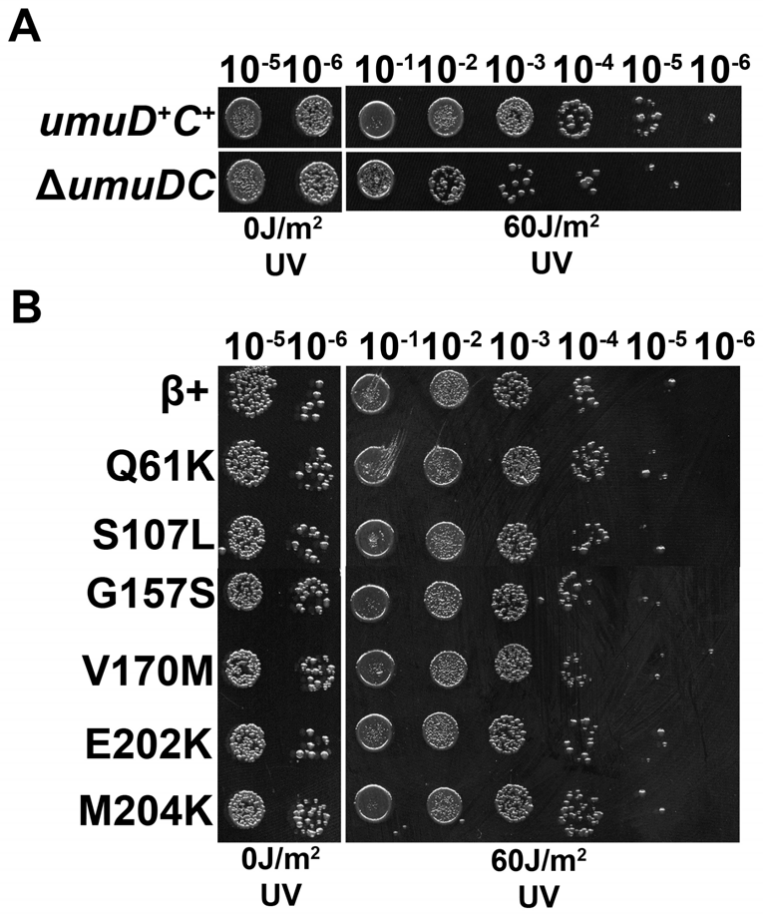
Ability of the mutant clamp strains to survive UV irradiation. (**A**) UV sensitivity of isogenic *dnaN^+^ umuD^+^C^+^* (RW118) and *dnaN^+^* Δ*umuDC* (RW120) strains was measured as described in *Materials and Methods*. (**B**) UV sensitivity of the *umuD^+^C^+^* plasmid shuffle strains MS202 (β^+^), MS203 (β^Q61K^), MS204 (β^S107L^), MS205 (β^G157S^), MS206 (β^V170M^), MS207 (β^E202K^) and MS208 (β^M204K^) was measured similarly. These experiments were performed in triplicate; results from one representative experiments are shown.

In addition to TLS, intact UmuD, together with UmuC, protects *E. coli* against UV-induced cell killing via a primitive DNA damage checkpoint [34]. In order to determine whether any of the mutant clamps were impaired for the UmuD_2_C checkpoint, we measured UV sensitivity of the different plasmid shuffle strains. As a control, we compared isogenic *umuD^+^C^+^* (RW118) and Δ*umuDC* (RW120) strains. As summarized in Fig. 4A, the *umuDC*-deficient strain was ∼10-fold more sensitive to UV than the isogenic *umuD^+^C^+^* strain, consistent with previous findings [34]. Based on results using the plasmid shuffle strains (Fig. 4B), each of the mutant clamps protected *E. coli* against cell killing by UV as well as the β^+^ control. These results, taken together with those discussed above, suggest the inability of these mutant clamps to impede growth at 30°C when co-overexpressed with Pol V is independent of the ability of β clamp to properly manage the TLS and checkpoint functions of *umuDC*.

### Overexpression of either β clamp or Pol V slows *E. coli* growth

The Zyskind lab determined that expression of β clamp at levels ∼12- to ∼38-fold above the normal physiological concentration blocked elongation of DNA replication [56]. Based on quantitative Western blot analysis, strains bearing plasmid pJRC210 express β clamp at ∼9-fold higher than physiological levels (see *Materials and Methods*). Thus, the level of β clamp expressed from pJRC210 under our experimental conditions is likely sufficient to interfere with replication. In light of these findings, we hypothesized the mutant clamps identified by virtue of their inability to impede growth at 30°C when co-overexpressed with Pol V may, in fact, have been selected because the mutations prevented clamp from impeding *E. coli* growth irrespective of Pol V function. As a test of this hypothesis, we examined the growth phenotype of strain AB1157 overexpressing only β^+^ from pJRC210 (without Pol V). As part of these experiments, we also analyzed growth of AB1157 overexpressing only Pol V (without β clamp).

**Figure 5:**
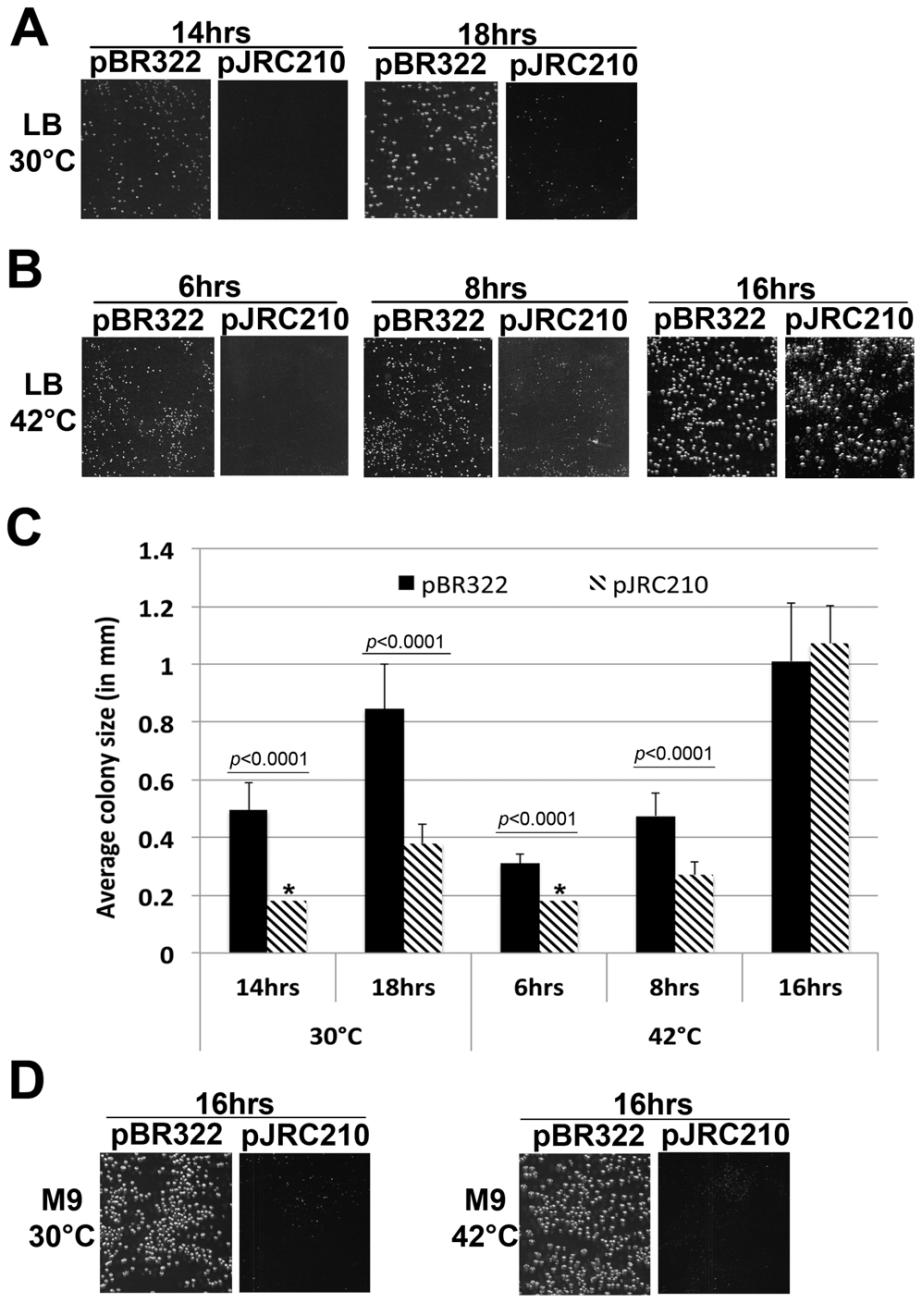
Effect of overexpression of β clamp on growth of AB1157. Representative images of LB agar plates showing pBR322 (control) or pJRC210 (β^+^) transformants of strain AB1157 following incubation at (**A**) 30° or (**B**) 42°C, as noted. (**C**) Diameters of representative CFUs shown in panels A and B were measured as described in *Materials and Methods*. The asterisk (*) indicates strains whose average colony diameter was below the measurement limit of 0.2 mm. Error bars represent one standard deviation. *P*-values ≤0.05 are indicated, and were calculated using the Student's *t*-test. (**D**) Shown are representative images of M9 agar plates of pBR322 (control) or pJRC210 (β^+^) transformants following incubation for 16 hrs at either 30° or 42°C. Colony diameters in panel D were not measured due to the small size of the pJRC210 transformants (*i.e*., diameters were <0.2 mm). Each transformation experiment was performed at least 3 independent times; results from one representative experiment are shown.

We first analyzed growth of AB1157 expressing elevated levels of β^+^. Since efforts to monitor growth of this strain in liquid culture gave inconsistent results, we followed growth of AB1157 following its transformation with either pBR322 (control) or pJRC210 (β^+^) on agar plates as a function of temperature and incubation time. As summarized in Fig. 5A & B, growth of AB1157 bearing pJRC210 lagged behind that of the same strain bearing pBR322 at both 30° and 42°C; Fig. 5C summarizes these results in quantitative form. We were unable to follow growth of the pJRC210 strain at 30°C beyond ∼18 hrs due to the accumulation of feeder colonies, which complicated analysis. However, after ∼16 hrs at 42°C, the size of the average AB1157 pJRC210 transformant was comparable to that of AB1157 bearing the pBR322 control plasmid (Fig. 5B & C). Taken together, these results indicate that ∼9-fold higher than normal physiological levels of β clamp slowed growth of *E. coli* irrespective of the incubation temperature. We also analyzed growth on M9 media. As summarized in Fig. 5D, the slow growth phenotype of the pJRC210 transformants was exacerbated on M9 agar compared to LB. Finally, pJRC210, but not pBR322, also slowed growth of *E. coli* strain MG1655 (V. M. P. Babu & M. D. Sutton, unpublished results), suggesting the slow growth phenotype was independent of the genetic background of the host strain.

**Figure 6:**
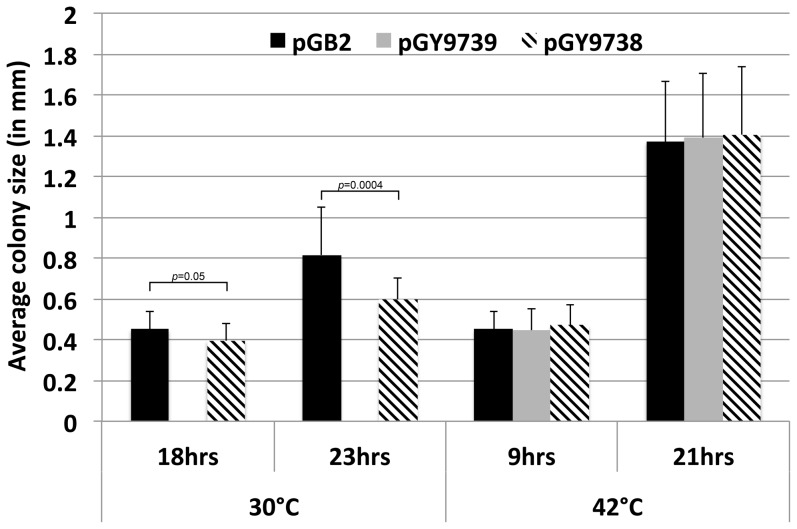
Effect of overexpression of the different *umuDC* gene products on growth of AB1157. Average colony diameters of pGB2 (control), pGY9739 (UmuD_2_C) or pGY9738 (UmuD'_2_C) transformants of strain AB1157 following growth at either 30°C or 42°C, as noted, are shown. No colonies were observed for the AB1157 pGY9739 transformant. Experiments were performed at least twice. Error bars represent one standard deviation. *P*-values ≤0.05 are indicated, and were calculated using the Student's *t*-test.

The growth phenotype of AB1157 expressing elevated levels of Pol V was examined similarly. For these experiments we used low copy number plasmids directing expression of either UmuD_2_C (pGY9739) or Pol V (UmuD'_2_C; pGY9738) from a mutant form of the *umuD^+^C^+^* promoter that bears the *o^c^_1_* mutation, which largely eliminates LexA repression [57], [58]. As a result, these plasmids express ∼6-fold higher than physiological levels of the different *umuDC* gene products in the absence of SOS induction [57]. Plasmid pGB2 was used as a negative control. Consistent with previous results [41]–[45], pGY9739 (UmuD_2_C) completely blocked growth of AB1157 at 30°C, but not 42°C (Fig. 6). This severe growth defect has been attributed to the checkpoint function of UmuD_2_C [41], [42], [44]. Transformation with pGY9738 (UmuD'_2_C) conferred a modest yet statistically significant growth defect at 30°C, but not 42°C, compared to the pGB2 control (Fig. 6). Taken together, these results indicate that, in addition to the well-documented ability of UmuD_2_C to impede *E. coli* growth [41]–[45], modest overexpression of either β clamp or Pol V alone also slows growth.

### Overexpression of mutant clamps fails to slow *E. coli* growth

**Figure 7:**
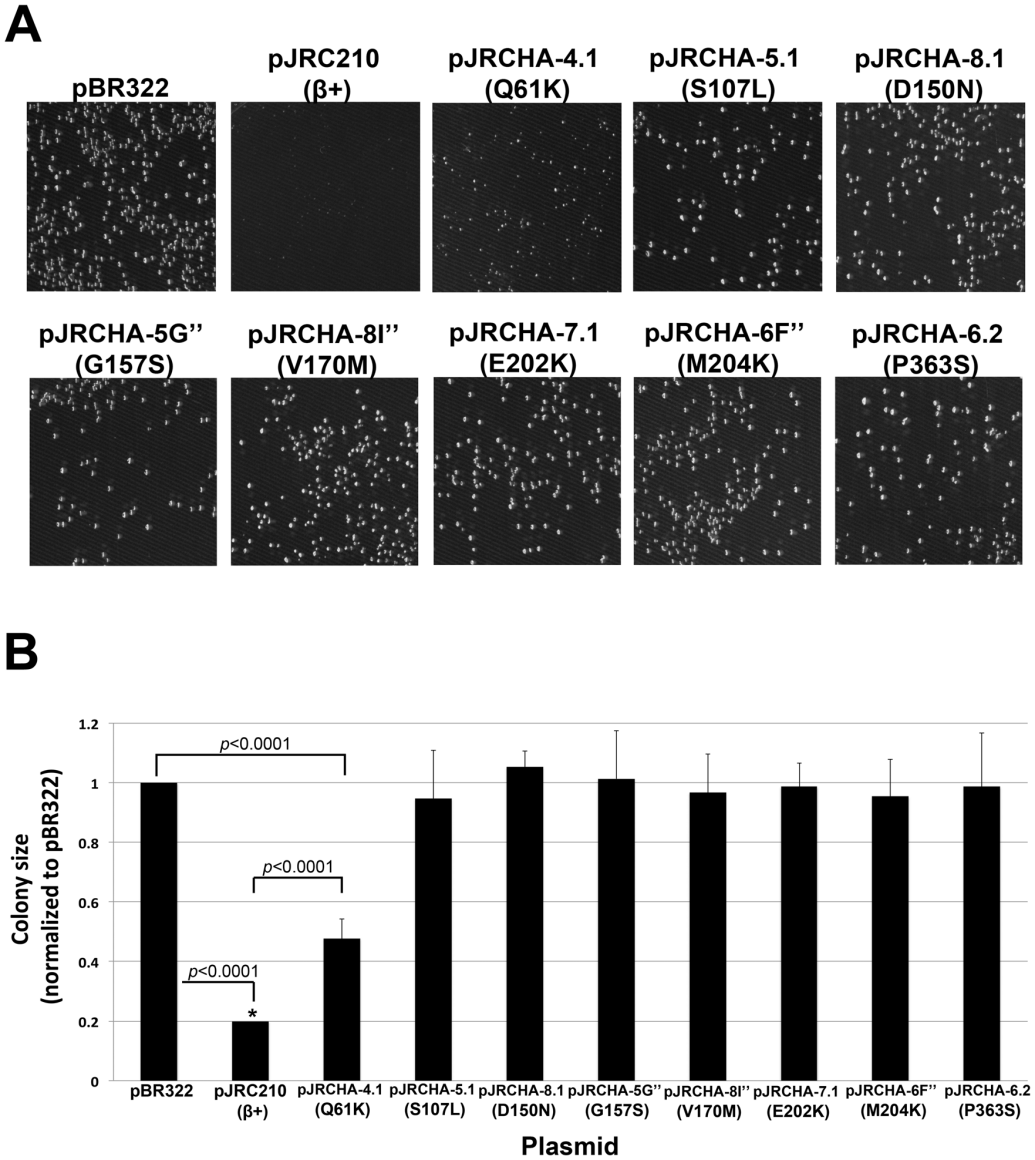
Effect of overexpression of different mutant clamps on growth of AB1157. (**A**) Shown are representative images of LB agar plates of AB1157 transformants following 18 hrs of growth at 30°C using the indicated plasmids. (**B**) Colonies were measured as described in *Materials and Methods*, and their respective sizes are represented relative to that observed for the AB1157(pBR322) control strain, which was set equal to 1.0. The asterisk (*) indicates strains whose average colony diameter was below the measurement limit of 0.2 mm. Experiments were performed at least twice. Error bars represent one standard deviation. *P*-values ≤0.05 are indicated, and were calculated using the Student's *t*-test.

In light of the findings discussed above, we asked whether overexpression of the different mutant β clamp proteins slowed growth of *E. coli*. With the exception of β^Q61K^, growth of AB1157 expressing the different mutant clamps closely mirrored the pBR322 control (Fig. 7), indicating they failed to slow growth. Although growth of the β^Q61K^ mutant was not as robust as the pBR322 control, it was nevertheless significantly more robust than AB1157 expressing the wild type clamp (Fig. 7). We previously demonstrated that each of these mutant clamp proteins was expressed at a level similar to that of the wild type clamp expressed from pJRC210 [44]. Thus, failure of these mutant clamps to slow *E. coli* growth is unrelated to their expression levels. Taken together, these results indicate that mutations in clamp that abrogate its ability to confer cold sensitive growth when co-overexpressed with Pol V [44] similarly alleviate the ability of ∼9-fold higher than normal physiological levels of the clamp to slow *E. coli* growth (see Fig. 7).

### Mutant clamps confer resistance to HU

**Figure 8:**
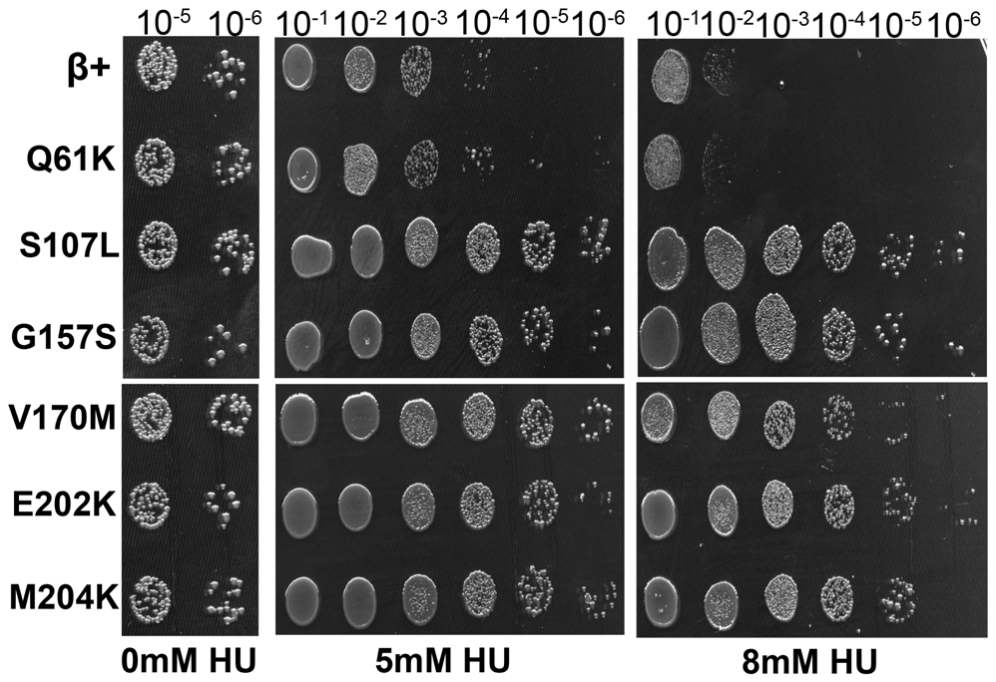
Mutant clamps confer resistance to HU. HU sensitivity was measured as described in *Material and Methods* using plasmid shuffle strains MS202 (β^+^), MS203 (β^Q61K^), MS204 (β^S107L^), MS205 (β^G157S^), MS206 (β^V170M^), MS207 (β^E202K^) and MS208 (β^M204K^). This experiment was performed at least twice; results from one representative experiment are shown.

We hypothesized that if the mutant clamps were identified in part because of their inability to perturb DNA replication, then strains expressing these mutant clamps should be more resistant to the lethal effects of replication fork stalling compared to the β^+^ control strain. As a test of this hypothesis, we used the plasmid shuffle strains to measure the level of HU sensitivity conferred by wild type or mutant clamps. HU stalls replication forks by inhibiting the *nrdAB*-encoded ribonucleotide reductase, effectively decreasing deoxyribonucleotide production, causing forks to stall [59], [60]. With the exception of β^Q61K^ (MS203), which resembled the β^+^ strain (MS202), each of the mutant clamps (MS204–MS208) conferred a significant level of HU^R^ (Fig. 8). The failure of β^Q61K^ to confer HU^R^ may relate to its intermediate phenotype regarding growth (Fig. 7). Regardless of the mechanism, these results support the hypothesis the mutant β clamp proteins are impaired for blocking elongation of DNA replication.

## Discussion

*E. coli* strains expressing ∼9-fold higher than normal physiological levels of the *E. coli* β clamp, together with ∼6-fold higher than normal SOS-induced levels of Pol V, fail to grow at 30°C [44]. Using a genetic assay that was independent of the ability of β clamp to support viability of *E. coli*, we previously described the isolation of 8 mutant β clamp proteins unable to impede growth when co-overexpressed with Pol V [44]. This growth defect was previously suggested to result from β clamp-Pol V interactions, which under conditions of their co-overexpression mimicked the checkpoint function of UmuD_2_C [44]. Importantly, this model has not yet been directly tested. The goal of this work was to better understand the relationship between the cold sensitivity conferred by co-overexpression of β clamp and Pol V, and the ability of these proteins to support DNA damage tolerance *in vivo*. While β^D150N^ and β^P363S^ failed to support *E*. *coli* viability, each of the remaining 6 mutant clamp proteins (β^Q61K^, β^S107L^, β^G157S^, β^V170M^, β^E202K^ and β^M204K^) supported normal growth when expressed as the only cellular clamp protein (Table 2). These same 6 mutants also supported Pol V-dependent UV- and MMS-induced mutagenesis *in vivo* (Fig. 3). Finally, each of these mutant clamps protected *E. coli* against cell killing by UV irradiation as well as the β^+^ control strain (Fig. 4). Taken together, these findings suggest the cold sensitivity conferred by co-overexpression of β clamp and Pol V is independent of the ability of clamp to manage the actions of Pol V in TLS and checkpoint. Consistent with this view, expression of β clamp at ∼9-fold higher than normal physiological levels slowed growth of *E. coli* AB1157 irrespective of the incubation temperature (Fig. 5). Moreover, growth of AB1157 was modestly slowed at 30°C by expression of Pol V ∼6-fold higher than normal SOS-induced levels (Fig. 6). Taken together, these findings support the conclusion that the combination of the individual effects on growth at 30°C conferred by elevated levels of β clamp and Pol V act to confer the severe cold sensitivity observed for the strain co-overexpressing these different proteins. Regardless of the mechanism, our ability to exploit the cold sensitive growth phenotype for direct selection of novel mutations in either β clamp, UmuD_2_C or Pol V with impaired function has unambiguously contributed to our understanding of structure-function relationships of these important and evolutionarily conserved proteins [6], .

We previously demonstrated that several of the mutant clamps were impaired for physical interactions with UmuD (β^V170M^ and β^P363S^) and/or UmuD' (β^G157S^ and β^P363S^) *in vitro*. However, this defect may not underlie the basis for their selection when co-overexpressed with Pol V; rather, this interaction defect may instead simply reflect the fact that several partners contact overlapping surfaces on clamp [10]–[12], [46], [61]. Indeed, results discussed in this report indicate the effect of the G157S and V170M mutations on the ability of clamp to manage the functions of *umuDC in vivo* was insignificant (P363S could not analyzed due to its inability to support *E. coli* viability). Furthermore, in addition to their reduced abilities to interact with UmuD/UmuD', each was additionally impaired for interactions with the catalytic subunit of the replicative Pol, Pol IIIα [46]. Thus, despite the fact that β clamp-Pol V interactions do not appear to serve as the mechanistic basis for the cold sensitive growth phenotype, it is not surprising that a subset of the clamp mutations identified nonetheless disrupt its interactions with one or both of the *umuD* gene products.

Remarkably, all 8 mutant clamps impaired for conferring cold sensitivity when co-overexpressed with Pol V were likewise impaired for slowing *E. coli* growth when expressed alone at ∼9-fold higher than normal physiological levels (Fig. 7). Our finding that elevated levels of β clamp slowed growth of *E*. *coli* is consistent with results from the Zyskind lab that ∼12-fold higher than physiological levels of β clamp interfered with elongation of DNA replication in *E*. *coli* [56]. Taken together, these findings suggest the slow growth phenotype we observed for the strain expressing ∼9-fold higher than normal levels of clamp was the result of impaired elongation. As noted above, β^G157S^, β^V170M^ and β^P363S^ were each impaired for interaction with Pol IIIα *in vitro* [46]. While the phenotypes of these 3 mutant clamps are consistent with the model that elevated levels of clamp interfere with elongation by sequestering Pol IIIα away from the replication fork, we would have expected all 8 clamp mutants to be impaired for interaction with Pol IIIα if this model were correct. That the remaining five β clamp mutants (β^Q61K^, β^S107L^, β^D150N^, β^E202K^ and β^M204K^) retained normal affinity for Pol IIIα *in vitro* [46] argues strongly that one or more alternative mechanisms contribute to the growth defect.

With the notable exception of β^Q61K^, each of the mutant clamp strains displayed significant resistance to HU (Fig. 8). Inasmuch as HU treatment acts to deplete cellular dNTPs by inhibiting catalytic activity of the *nrdAB*-encoded ribonucleotide reductase, these results suggest the mutant clamps were less sensitive to replisome stalling. This phenotype is consistent with the idea that elevated levels of β clamp act to slow *E. coli* growth by perturbing DNA replication. Regardless of the mechanism, the HU^R^ phenotype conferred by all but β^Q61K^ is consistent with these mutant clamps failing to arrest elongation. We are currently analyzing these mutant clamps biochemically to define the molecular basis for their HU^R^ phenotype.

In addition to providing insight into why co-overexpression of β clamp and Pol V impedes *E. coli* growth at 30°C, results discussed in this report also revealed that residues D150 and P363 of β clamp perform one or more functions critical to the viability of *E*. *coli* (Table 2). We previously demonstrated that both of these mutant clamps complemented the temperature sensitive growth phenotype of the *dnaN159*(Ts) strain [10]. Taken together, these findings suggest β^D150^ and β^P363^ are functional as heterodimers with β159 *in vivo*; this implies viability relies on just one of the two clamp protomers lacking mutations at these positions. Importantly, position P363 is located near the C-terminus of β clamp, and forms part of the hydrophobic cleft that is contacted by the CBM present in the different clamp partner proteins ([8], [62]; see Fig. 1). Substitution of P363 with serine in β^P363S^ impaired interaction of clamp with Pol IIIα *in vitro* [46], possibly explaining why this mutant clamp cannot support *E. coli* viability. Interestingly, β159 bears a G174A substitution that also affects the clamp cleft (see Fig. 1). However, β bearing only the G174A mutation (β781) supports *E. coli* viability, and fails to confer temperature sensitive growth [12]. These findings, taken together with our observation that β^P363S^ can complement the *dnaN159*(Ts) strain [10], suggests the G174A substitution confers a modest effect on the clamp cleft compared to P363S, at least with respect to clamp functions required for *E. coli* viability.

Position D150 of β clamp is located in a large solvent exposed loop known to interact with the DNA template ([5], [6]; labeled loop ‘2’ in Fig. 1), as well as TLS Pols II, IV and V [6]. We previously demonstrated that a mutant clamp bearing alanine substitution of residues H148-R152 (β^148–152^) within this loop failed to support *E. coli* viability when crossed onto the bacterial chromosome [6]; a similar result was observed for this same clamp mutant using the plasmid shuffle assay (Table 2; see pACMβ5A). Thus, failure of β^148–152^ to support *E. coli* viability may be attributable to the loss of function conferred by substitution of D150 with Asn. In theory, the D150 side chain can interact with the proton on N4 of cytosine [63]. However, position D150 of clamp did not contact the DNA template in the x-ray crystal structure of clamp on DNA solved by Georgescu *et al*., despite the fact that the DNA template used contained five cytosine residues [5]. Although a DNA binding defect of β^D150N^ might underlie its failure to support *E. coli* viability, a less direct role in DNA binding is also possible. Position Q149 of clamp is postulated to sense the presence of DNA within the central pore of the clamp and relay this information to D150, which, in turn, contacts R152 [64]. Position R152 additionally contacts both DNA and the δ subunit of the DnaX clamp loader [5], [6], [62]. Thus, β^D150N^ may be impaired for relaying the status of clamp-DNA interactions to other parts of the protein and/or to δ/DnaX, thereby interfering with clamp loading. Such a defect could explain the inability of β^D150N^ to support growth of *E. coli*.

In summary, results discussed in this report extend our understanding of the role of the β sliding clamp in *umuDC*-mediated cold sensitivity. Specifically, they support the model that cold sensitivity is due, at least in part, to the combination of the individual effects conferred by clamp and Pol V on growth at 30°C. Moreover, results discussed above suggest the mutant clamps were identified due to their inability to slow growth rather than an inability to interact with Pol V. In addition, our findings discussed above reveal the practicality of our *dnaN* plasmid shuffle assay (see Fig. 2). To date, efforts to define mutant *dnaN* phenotypes *in vivo* have focused on measuring the ability of ectopically expressed mutant β proteins to complement phenotypes of the thermolabile *dnaN159*(Ts) strain (*e*.g., see [10], [18], [61]). We recently determined that β159 forms heterodimers with other β clamp proteins *in vivo*, and that such heterodimers can support *E. coli* viability at 42°C regardless of whether the ectopically expressed clamp protein retains function [6], [49]. As a result, observed phenotypes are a reflection of a heterodimeric form of clamp rather than the homodimeric mutant [6], [49]. The *dnaN* plasmid shuffle assay described in this report will not only circumvent this issue, but also provides a rapid and simple assay with which to identify and characterize new *dnaN* alleles with novel phenotypes, helping to refine our understanding of β clamp structure-function relationships.
